# The impact of automatic speech recognition technology on second language pronunciation and speaking skills of EFL learners: a mixed methods investigation

**DOI:** 10.3389/fpsyg.2023.1210187

**Published:** 2023-08-16

**Authors:** Weina Sun

**Affiliations:** School of Foreign Languages, Changchun Institute of Technology, Changchun, China

**Keywords:** automatic speech recognition, L2 pronunciation, speaking, EFL, accentedness, comprehensibility, mixed methods

## Abstract

**Introduction:**

This study employed an explanatory sequential design to examine the impact of utilizing automatic speech recognition technology (ASR) with peer correction on the improvement of second language (L2) pronunciation and speaking skills among English as a Foreign Language (EFL) learners. The aim was to assess whether this approach could be an effective tool for enhancing L2 pronunciation and speaking abilities in comparison to traditional teacher-led feedback and instruction.

**Methods:**

A total of 61 intermediate-level Chinese EFL learners were randomly assigned to either a control group (CG) or an experimental group (EG). The CG received conventional teacher-led feedback and instruction, while the EG used ASR technology with peer correction. Data collection involved read-aloud tasks, spontaneous conversations, and IELTS speaking tests to evaluate L2 pronunciation and speaking skills. Additionally, semi-structured interviews were conducted with a subset of the participants to explore their perceptions of the ASR technology and its impact on their language learning experience.

**Results:**

The quantitative analysis of the collected data demonstrated that the EG outperformed the CG in all measures of L2 pronunciation, including accentedness and comprehensibility. Furthermore, the EG exhibited significant improvements in global speaking skill compared to the CG. The qualitative analysis of the interviews revealed that the majority of the participants in the EG found the ASR technology to be beneficial in enhancing their L2 pronunciation and speaking abilities.

**Discussion:**

The results of this study suggest that the utilization of ASR technology with peer correction can be a potent approach in enhancing L2 pronunciation and speaking skills among EFL learners. The improved performance of the EG compared to the CG in pronunciation and speaking tasks demonstrates the potential of incorporating ASR technology into language learning environments. Additionally, the positive feedback from the participants in the EG underscores the value of using ASR technology as a supportive tool in language learning classrooms.

## Introduction

According to research, the ability to communicate in a foreign language is closely related to the individual’s qualification in pronunciation (Thomson and Derwing, 2015; Evers and Chen, 2022). Pronunciation accuracy influences not just language instructors but also learners’ self-assurance and job prospects (e.g., Hosoda et al., 2012). Computer-Assisted Pronunciation Training (CAPT) has been employed in EFL instruction to assist students in improving their pronunciation (Neri et al., 2008). CAPT technologies seek to provide language students with personalized, multimodal, digital-based pronunciation training (Thomson, 2011; Evers and Chen, 2022). Notwithstanding their shown efficacy, certain CAPT technologies may be challenging for trainees to use (Levis, 2007) and might confine their education to pre-planned behaviors (Neri et al., 2008; McCrocklin, 2019a). Computers can provide language learners with greater freedom from classrooms by allowing them to concentrate on their educational materials at almost any moment of the day (Elimat and AbuSeileek, 2014; Peng et al., 2021; Lei et al., 2022). Lee (2000) lists several reasons for integrating computer technology into language instruction, including: (1) the potential for greater motivation among students; (2) improved academic performance; (3) access to more authentic learning materials; (4) increased collaboration among learners; and (5) the ability to repeat lessons as many times as necessary.

Many language instructors strive to incorporate the fundamental grammar, vocabulary, culture, and practice of the four language skills into their sessions with less emphasis on pronunciation teaching (Munro and Derwing, 2011; Elimat and AbuSeileek, 2014). As noted by Lord (2008), a lot of language instructors believe that by providing additional instruction in the second language, pupils would learn to pronounce it on their own; other language instructors question whether it is essential to provide instruction in the segmental and supra-segmental phonological aspects of a language (Thomson and Derwing, 2015). However, numerous strategies, such as corrective feedback and complete immersion learning, are brought to pronunciation training by technology devices (Eskenazi, 1999; Luo, 2016; Tseng et al., 2022), leading to a development that goes beyond the boundaries of the classroom and allows students to have freedom and control over their language learning abilities (Pennington, 1999; Thomson, 2011; O’Brien et al., 2018).

Automatic speech recognition (ASR) technology, as a type of CAPT system, is a speech-decoding and transcription technique that enables learners to independently study any topic (Levis and Suvorov, 2014). ASR is a technology that enables computers to convert human speech into text. ASR has been developed using various techniques, including statistical models, machine learning, and neural networks (Loukina et al., 2017; Inceoglu et al., 2020). These models are trained on large datasets of spoken language, which enables them to recognize and transcribe speech accurately (Ahn and Lee, 2016). ASR technology has a wide range of applications, from dictation and transcription services to virtual assistants and language learning (Chiu et al., 2007; Yu and Deng, 2016).

Advanced ASR systems have the ability to offer feedback on the sentence, word, or textual level (Elimat and AbuSeileek, 2014; McCrocklin, 2019a). Automated feedback can range from rejecting weakly pronounced statements to detecting particular problems in phonetic clarity or phrase accent (Yu and Deng, 2016; Lai and Chen, 2022). This feedback can make the students aware of challenges with their pronunciation, which is the first step toward resolving these issues, and it may also help learners avoid acquiring bad speech patterns (Eskenazi, 1999; Elimat and AbuSeileek, 2014; Wang and Young, 2015). Because instructors in typical language teaching situations have limited opportunity to complete assessments and offer individual feedback (Nguyen and Newton, 2020; Liu et al., 2022), the ability to accomplish these activities automatically is seen as one of the primary benefits of ASR-based learning (Neri et al., 2002; Jiang et al., 2023). Notwithstanding its expanding prominence and benefits, limited research has been conducted to evaluate the influence of ASR technology on FL learners’ speaking abilities in educational contexts (Jiang et al., 2021; Inceoglu et al., 2023), especially in the Chinese EFL context. In other words, there has been limited research on the utility of ASR technology in enhancing L2 pronunciation and speaking skill of EFL learners (Garcia et al., 2020; Dai and Wu, 2021). Due to the importance of correct and comprehensible pronunciation in communication and the positive effects of ASR systems in enhancing speech (Kim, 2006; Evers and Chen, 2022), the present study used an explanatory sequential design to investigate the effect of ASR technology in improving the pronunciation and speaking skills of EFL learners. This study provides valuable insights into the potential of ASR technology with peer correction for enhancing L2 pronunciation and speaking skills in Chinese EFL learners, and highlights its potential as a tool for language learning in the future. This study was guided by the following research questions:

1. Does the use of automatic speech recognition technology (ASR) with peer correction lead to improvements in L2 pronunciation and speaking skills compared to traditional teacher-led feedback and instruction?
2. What are the perceptions of EFL learners regarding the usefulness of ASR technology in improving their L2 pronunciation and speaking skills?

## Literature review

### Theoretical background

The theoretical framework underpinning this research is rooted in Vygotsky’s Socio-Cultural Theory (SCT) (Vygotsky, 1978), which emphasizes the pivotal role of social interaction and context in language learning. SCT recognizes the significance of social interactions and cultural context in shaping cognitive development and learning processes. Within the domain of L2 learning, SCT posits that learners acquire L2 skills through meaningful interactions with proficient speakers of the target language (Lantolf, 2006). In this study, ASR technology with peer correction was employed as a pedagogical tool for language learning, aligning with the principles of SCT (Jiang et al., 2021). The utilization of ASR technology and the integration of peer correction aim to foster an interactive and engaging learning environment for EFL learners, providing them with valuable feedback on their pronunciation and facilitating the acquisition of enhanced L2 pronunciation and speaking skills (Xiao and Park, 2021). The inclusion of peer correction further amplifies the social dimension of the learning process, enabling learners to engage with their peers, exchange knowledge, and collaborate in the pursuit of improving their pronunciation and speaking abilities. Furthermore, SCT recognizes language learning as a dynamic process, influenced continuously by social, cultural, and environmental factors (Vygotsky, 1986). By incorporating ASR technology, learners are presented with opportunities for authentic and meaningful communication, which may stimulate their motivation and engagement in the learning process, contributing to their overall language development.

### The importance of pronunciation in L2

The importance of pronunciation in L2 acquisition is often overlooked in classroom instruction (Derwing and Munro, 2015). However, research has established a direct link between a speaker’s pronunciation proficiency and their overall L2 communication competence (Offerman and Olson, 2016; Bashori et al., 2022). To facilitate effective pronunciation acquisition, timely and personalized feedback is crucial after students deliver their utterances (Cucchiarini et al., 2012). Yet, providing individualized remedial feedback to all students by FL instructors can be arduous, expensive, and impractical, particularly within the L2 classroom setting (Cucchiarini et al., 2012). It requires not only an understanding of specific sounds but also the ability to produce those sounds, which necessitates significant teaching and feedback efforts (McCrocklin, 2016).

Recent advancements in technology have demonstrated that ASR technology can be an effective tool for enhancing the speaking abilities of FL learners (Evers and Chen, 2021; Bashori et al., 2022). Chen (2017) reported that a majority of learners find ASR-based websites helpful and that these websites can assist students in improving their English-speaking skills. Golonka et al. (2014), in their review of research, noted that while ASR reliability is not perfect, students often have positive experiences when using ASR-based programs. Furthermore, Cucchiarini et al. (2009) found that although the developed ASR-based system did not identify all errors made by students, the feedback provided was beneficial in helping learners improve their pronunciation after a short period of practice. Via leveraging ASR technology, language learners can receive valuable and personalized feedback on their pronunciation, which can contribute to their overall speaking proficiency (Bashori et al., 2022). This emerging technology offers a promising avenue for addressing the challenges associated with providing individualized pronunciation instruction within the FL classroom (Chiu et al., 2007). Although it is acknowledged that error rates in ASR technology still pose challenges, Morton et al. (2012) have highlighted this concern. Nonetheless, employing spoken activities through computer-based platforms has been found to foster increased motivation and engagement in speaking tasks in foreign or second language learning contexts, as emphasized by Golonka et al. (2014). Additionally, Luo (2016) conducted a study that demonstrated the effectiveness of integrating CAPT in reducing students’ pronunciation difficulties to a greater extent compared to traditional in-class training methods.

### Pronunciation training

Pronunciation training plays a crucial role in facilitating comprehension of L2 dialog (Offerman and Olson, 2016) and mitigating the risk of miscommunication for students with poor pronunciation (Evers and Chen, 2021). While pronunciation and grammar issues can impact message comprehension to some extent, they do not entirely impede understanding (Crowther et al., 2015). Therefore, effective pronunciation training can significantly enhance students’ communication skills and overall satisfaction with their EFL courses. However, pronunciation improvement is often undervalued and seen as a non-essential objective, consuming precious instructional time in the classroom (Gilakjani and Ahmadi, 2011).

Moreover, many instructors feel ill-equipped to provide pronunciation education due to the perception that it requires specialized training (Couper, 2017; Evers and Chen, 2021). Couper (2017) found that a lack of knowledge about pronunciation training among 19 EFL instructors in New Zealand led to uncertainty about how to address students’ poor pronunciation and what aspects to prioritize. Insufficient training in phonology contributed to their limited understanding of the phonological processes underlying sound production, stress, rhythm, and intonation. Consequently, these instructors faced challenges in effectively explaining these complex processes to students, which is crucial for efficient pronunciation instruction (Baker and Burri, 2016). Additionally, non-native language instructors often lack confidence in their own pronunciation due to their background (Jenkins, 2007). Despite these limitations, research suggests that pronunciation training can enhance student learning (Lee et al., 2015) and improve the comprehensibility of L2 output (Offerman and Olson, 2016). However, Benzies (2013) study reveals that students perceive current pronunciation activities, primarily focused on listening and repeating, as monotonous. Overall, pronunciation training is an essential aspect of language instruction, yet it is often undervalued and teachers may feel inadequately prepared to address students’ pronunciation difficulties. However, research demonstrates the positive impact of pronunciation training on student learning and comprehensibility.

### ASR and pronunciation training

In response to the challenges of pronunciation enhancement, researchers have turned to technology as a potential solution. Integrating technology into pronunciation activities has been found to decrease L2 learners’ anxiety and create a more conducive learning environment (Nakazawa, 2012). Technology, such as Computer-Assisted Pronunciation Training (CAPT), offers features that traditional classrooms may lack, including ample practice time, stability, objective feedback, and visual representations (Levis, 2007).

One key advantage of technology-based feedback, particularly in CAPT, is its ability to adapt to individual learning styles and needs, which is often difficult to achieve in traditional classrooms (Mason and Bruning, 2001). Moreover, CAPT systems provide real-time feedback, allowing both learners and teachers to address current difficulties promptly without interrupting the speaking process (Mason and Bruning, 2001). However, despite the benefits of CAPT in speech improvement, some systems may inadvertently limit learners’ practice opportunities (Evers and Chen, 2021). CAPT technologies that rely on visual feedback, such as spectrograms or waveforms, often require pre-recorded native speaker samples or predetermined phrases for comparison (McCrocklin, 2019b). These systems can be technologically challenging for both learners (Wang and Young, 2015; Garcia et al., 2018) and instructors (Neri et al., 2008). For example, participants in Wang and Young’s (2015) study expressed difficulty in understanding waveform graphs, highlighting the complexity of visual representations as a primary barrier to learners’ progress. Neri et al. (2002) found that initial training on the interface and familiarization with feedback representations and interpretation significantly consumed valuable class time.

The focus on dictation ASR software for language acquisition, despite its original design purpose, has gained traction among researchers (Liakin et al., 2017). This type of software, often available for free, benefits from a large voice database, resulting in improved decoding quality. Additionally, its accessibility allows for quick deployment in classrooms or self-study exercises (Evers and Chen, 2021). ASR systems can be classified into three types: speaker-dependent, speaker-independent, and speaker-adaptive. Speaker-dependent ASR requires input from the user to train the system to recognize their speech characteristics. Speaker-independent ASR, on the other hand, operates without prior training by utilizing a vast speech database. Speaker-adaptive ASR employs customized speech databases adapted to each user through specific algorithms. Among these types, speaker-independent ASR is most suitable for teaching accurate pronunciation, as it avoids becoming accustomed to the speaker’s accent or mispronunciations (Kitzing et al., 2009; Ding et al., 2022).

ASR dictation software is considered enjoyable, helpful, and user-friendly (Mroz, 2018). It serves as an effective tool for students to practice pronunciation and detect common errors (McCrocklin, 2016). However, the flexibility of interacting with ASR dictation systems comes at the expense of output accuracy. Some speech recognition software, such as the Google Speech Recognition engine, achieves high accuracy, accurately interpreting 93% of non-native free speech (McCrocklin, 2019a). In contrast, other software, such as Windows Speech Recognition or Siri, provides lower accuracy, with Windows Speech Recognition decoding 74% and Siri decoding 69% correctly (Daniels and Iwago, 2017). Inaccurate voice transcription can lead to frustration and reduced enthusiasm among students. McCrocklin (2019a) reported in their qualitative study that several participants expressed concerns about the reliability of ASR software in their pronunciation practice.

Despite its limitations, various studies have demonstrated the usefulness of available ASR software in L2 speech instruction and pronunciation improvement (Cucchiarini et al., 2009; McCrocklin, 2019a,b; Garcia et al., 2020; Inceoglu et al., 2020, 2023; Evers and Chen, 2021, 2022; Yenkimaleki et al., 2021; Cámara-Arenas et al., 2023). For example, Liakin et al. (2015) compared ASR, instructor-based pronunciation feedback, and no feedback techniques and found that only the ASR strategy significantly improved students’ pronunciation. McCrocklin’s (2016) experimental investigation revealed substantial improvements in students’ pronunciation even after a short period of ASR instruction. Similarly, Mroz (2018) discovered that practicing with ASR positively impacted students’ pronunciation.

Furthermore, ASR dictation systems have proven beneficial in studies involving Chinese-speaking students, leading to improvements in English pronunciation Liu et al., 2019. ASR software meets the selection criteria for pronunciation software outlined by Chapelle and Jamieson (2008), as it fulfills students’ requirements, provides explicit instruction, offers opportunities for students to practice and evaluate their technology-supported speech, delivers intelligible and accurate feedback, and fosters independent learning (Liakin et al., 2015).

## The present study

As previously mentioned, the extant literature provides substantial evidence regarding the importance of L2 pronunciation and speaking skills in EFL learning contexts. However, there are challenges faced by L2 learners in acquiring accurate pronunciation and fluent speaking abilities, as well as the significant impact these skills have on overall language proficiency (Isaacs, 2018). In response to these challenges, researchers and educators have explored various approaches to enhance L2 pronunciation and speaking instruction. One promising avenue that has emerged is the integration of ASR technology into language learning environments. ASR technology offers learners the opportunity to receive immediate and objective feedback on their pronunciation, allowing for targeted practice and self-assessment (Inceoglu et al., 2020, 2023; Cámara-Arenas et al., 2023). Additionally, the use of ASR technology with peer correction brings a social component to the learning process, fostering collaboration, interaction, and shared knowledge among learners (McCrocklin, 2019a,b).

The present study has distinct characteristics that set it apart from similar investigations. It employs a mixed methods approach, combining quantitative and qualitative data collection and analysis techniques to comprehensively understand the influence of ASR technology on L2 pronunciation and speaking skills in EFL learners. By integrating objective measurements and subjective learner perceptions, the study captures a multifaceted perspective. Specifically, it focuses on the integration of ASR technology with peer correction in L2 pronunciation and speaking instruction, uncovering unique outcomes from their combined impact. This study fills a gap by exploring the viewpoints of intermediate-level Chinese EFL learners regarding their experience, contributing insights to the existing research. The study employs a diverse range of assessment measures, including read-aloud tasks, spontaneous conversations, and IELTS speaking tests, ensuring a comprehensive evaluation of learners’ spoken performance in controlled and spontaneous contexts. The findings offer a nuanced understanding of the effects of ASR technology with peer correction on various dimensions of L2 pronunciation and speaking skills.

## Methods

This study utilized an explanatory sequential mixed methods design (Creswell et al., 2004), which involves the collection and analysis of both quantitative and qualitative data in a sequenced manner. The initial quantitative phase was conducted to determine the effectiveness of ASR technology with peer correction in enhancing L2 pronunciation and speaking skills in Chinese EFL learners. The subsequent qualitative phase was added to explore the students’ perceptions of the ASR technology and gain a deeper understanding of how the technology impacted their learning experience. This design allows for a comprehensive analysis of the research question by integrating both quantitative and qualitative data, providing a more nuanced understanding of the phenomenon under investigation.

## Participants

The study was conducted at a language training center in Shenzhen, Guangdong province, China, with the approval of the center’s authorities and informed consent of the participants. The sample consisted of 61 Chinese EFL learners with an age range of 20 to 31 years (*M* = 25.5, SD = 3.6), of which 52% (*n* = 32) were male and 48% (*n* = 29) were female. The participants were enrolled in a 14-week English pronunciation course, with two classes randomly assigned to two different research conditions. One class consisting of 32 students served as the control group and received traditional teacher-led feedback and instruction, while the other class consisting of 29 students was the experimental group and used automatic speech recognition technology (ASR) with peer correction. All participants were native Chinese speakers and had no prior experience studying abroad. Their English proficiency level was intermediate (B1 level in the Common European Framework of Reference for Languages), as assessed by their English language scores on the college entrance examinations, which are administered once a year in China. An independent-samples t-test showed that no significant difference was found in English proficiency level between the control and experimental groups, *t* (59) = −0.85, *p* = 0.399.

An experienced professional male IELTS teacher, who collaborated closely with the researchers, served as the instructor responsible for delivering the intervention and providing feedback to the participants of both groups. The researcher’s primary role was to monitor and oversee the intervention process, ensuring its adherence to the research design and objectives. The researcher worked closely with the instructor to design the intervention protocol, develop the assessment measures, and ensure consistency across groups. To enhance the credibility and objectivity of the study, the researcher maintained a reflexive stance throughout the research process. Regular meetings and discussions were held with the instructor to align interpretations and ensure consensus in data analysis and theme development.

## Procedure

During each lesson, the intervention and control groups followed a structured protocol to target L2 pronunciation skills. The intervention utilized the “Speechnotes - Speech to Text” dictation ASR software, which was accessed by the experimental group (EG) through their individual laptops and a dedicated website. Prior to commencing the intervention, the teacher provided a comprehensive demonstration of the ASR website interface, highlighting its functionalities and demonstrating how learners could interpret the software’s feedback effectively.

To initiate the intervention, the participants were divided into small groups consisting of three or four individuals in both the EG and control group (CG). Each group received a carefully selected text, specifically designed to address common pronunciation challenges encountered by Chinese EFL learners. The text, comprising approximately 150–200 words, incorporated a range of phonetic patterns and problematic sounds identified from previous studies (Zhang and Yin, 2009). These linguistic features aimed to ensure that the intervention targeted the learners’ specific needs.

During the reading stage, one student assumed the role of the practicing student (PS) within each small group. The PS took turns reading a paragraph aloud while the other group members actively listened and provided focused attention to the PS’s pronunciation and intonation. In the EG, the Speechnotes ASR software was utilized to transcribe the PS’s reading in real-time. The software, powered by the Google Speech Recognition engine, boasted an accuracy rate exceeding 90%, as reported by the developers. Meanwhile, in the CG, the teacher directly provided pronunciation feedback to the PS, employing strategies such as modeling correct pronunciation, offering explicit explanations, and suggesting specific improvement techniques.

In the subsequent pronunciation feedback stage, the EG members meticulously reviewed the ASR software’s transcription, comparing it to the original text. They conscientiously marked any incorrectly identified words or phrases on a printed transcription. This collaborative process encouraged active engagement and critical evaluation of the ASR output, fostering metalinguistic awareness among the learners. Conversely, the CG participants received personalized pronunciation feedback from the teacher, who carefully analyzed their performances and provided constructive comments and guidance for improvement.

In the practice stage, the EG’s team members worked collaboratively to address the misidentified words and phrases identified in the ASR software’s transcription. They leveraged the software’s feedback, the highlighted transcription, and their collective knowledge to correct the mispronunciations. This collaborative problem-solving approach facilitated peer learning and encouraged the development of self-correction skills. The CG, on the other hand, individually focused on implementing the teacher’s feedback, engaging in targeted pronunciation practice without direct peer support.

To ensure consistency and comparability across all groups, team members in both the EG and CG engaged in pronunciation-based discussions during the practice stage. These discussions aimed to enhance learners’ metacognitive abilities and foster a deeper understanding of pronunciation principles. Participants in each small group assessed and ranked each other’s pronunciation performances, providing constructive feedback and suggesting specific strategies for improvement. The discussions also created a supportive and motivating environment for learners to reflect on their own pronunciation and gain insights from their peers’ perspectives.

After each team member completed reading the assigned text, the role of the PS was rotated, ensuring that every learner had an opportunity to practice and receive feedback from their peers and the teacher. This systematic rotation facilitated equal participation and ensured that the benefits of the intervention were distributed evenly among the learners.

It is worth noting that the final 40 min of each lesson were dedicated to general English tasks, unrelated to pronunciation, which were carefully designed to maintain consistency and control between the EG and CG. These tasks encompassed various language skills, such as reading comprehension, vocabulary acquisition, and grammar exercises.

Also, ethical considerations were carefully addressed throughout the research process to ensure the well-being and rights of the participants. This study obtained ethical clearance from the relevant institutional review board before data collection commenced. Informed consent was obtained from all participants, who were provided with detailed information regarding the purpose of the study, their rights as participants, and the voluntary nature of their participation. Participants were assured of the confidentiality and anonymity of their data, and their right to withdraw from the study at any time without penalty.

To protect the participants’ privacy, all data collected were securely stored and accessible only to the research team. Identifying information was removed or anonymized to ensure confidentiality. Data were analyzed and reported in aggregate form to prevent the identification of individual participants. Pseudonyms or participant codes were used in reporting qualitative findings to further protect participant anonymity.

## Instruments

### Pronunciation measures

The initial tool used for evaluating the students’ pronunciation was a read-aloud task, which has been employed in previous studies (e.g., Thomson, 2011). The reading activity involved seven sentences that aimed to assess seven English phonemes that are known to pose challenges for Chinese speakers (Zhang and Yin, 2009), as well as the accuracy of stress, juncture, and intonation within sentences. Throughout the task, two instructors evaluated the students’ performance using a 9-point scale for accentedness (ranging from 1, indicating heavily accented, to 9, indicating native-like) and comprehensibility (ranging from 1, indicating very difficult to understand, to 9, indicating no effort required to understand).

Also, following Evers and Chen (2022), the researcher used spontaneous conversation to measure participants’ pronunciation. To assess the participants’ pronunciation in spontaneous conversation, a short conversation including three to four questions was used as the second instrument. The assessment was based on the IELTS speaking skill rubric, which is a widely recognized and standardized assessment tool. The rubric rates pronunciation on a 9-point scale, with each point corresponding to a specific description of the level of pronunciation proficiency. Spontaneous speech allows students to freely choose their words and expressions, and may reveal pronunciation difficulties that were not apparent in the reading aloud task. However, it also requires students to focus on conveying meaning, which can distract them from their pronunciation. In contrast, the reading aloud task focuses solely on pronunciation, allowing for more accurate assessment of the pronunciation of certain sounds and words. Using both the reading aloud task and spontaneous conversation can provide a comprehensive assessment of the participants’ pronunciation abilities.

### The IELTS speaking skill test

The IELTS Speaking Skill Test is a comprehensive assessment tool that evaluates learners’ speaking ability in four areas: fluency and coherence, lexical resources, grammatical range and accuracy, and pronunciation. Each of the four criteria is given equal weight, and participants are scored on a scale from 1 to 9 for each part of the test. These scores are then combined and divided by four to obtain a mean score, which serves as the participant’s overall band score. The assessment is conducted in an interview format and covers general information questions, topic description, and topic discussion. To guarantee impartiality and diminish partiality, every participant was evaluated and graded by two skilled and debriefed evaluators, one of whom is a researcher, and inter-rater consistency is evaluated using Kendall’s tau-b coefficient. The inter-rater reliability analysis yielded a Kendall’s tau-b coefficient of 0.87 for the evaluation of participants’ speaking performance. This coefficient indicates a high level of agreement and consistency between the two evaluators’ ratings. The obtained value suggests a strong positive correlation between the scores assigned by the evaluators, demonstrating their shared understanding and alignment in assessing the participants’ speaking abilities.

### Semi-structured interview

The data for the qualitative phase was collected through semi-structured interviews (See the Appendix). The interviews were conducted with seven volunteer participants in person or via video conferencing, depending on the participants’ preferences. The interview guide was designed to explore participants’ perceptions of the effectiveness of ASR technology for improving their pronunciation, their motivation and engagement in using the technology, and the specific benefits they experienced from using the technology.

The qualitative data collection in this study involved conducting semi-structured interviews with seven volunteer participants to gain in-depth insights into their perceptions and experiences. The interviews were conducted either in person or via video conferencing, based on the participants’ preferences and logistical considerations. The interview guide, which can be found in the Appendix, was carefully designed to explore various aspects related to the effectiveness of ASR technology in enhancing pronunciation skills, as well as the participants’ motivation, engagement, and specific benefits derived from utilizing the technology.

During the interviews, participants were provided with a comfortable and supportive environment to freely express their thoughts and experiences. The researcher followed a semi-structured approach, allowing for flexibility and probing into relevant areas while ensuring that the key research questions and themes were addressed. The interviews were audio-recorded with the participants’ consent to ensure accurate data capture and subsequent analysis.

The timing of the interviews was strategically planned in reference to the intervention phase. Specifically, the interviews were conducted after the completion of the intervention to allow participants sufficient exposure and engagement with the ASR technology and peer correction activities. This enabled them to reflect on their experiences and provide valuable insights into the impact and effectiveness of the intervention on their pronunciation and speaking skills.

### Data analysis

To ensure the accuracy of the pronunciation test scores comparison, the rating reliability of the two evaluators was first assessed using the intraclass correlation coefficient (ICC). Our analysis indicated a high level of agreement between the evaluators for the reading and spontaneous conversation pre- and post-tests. Specifically, for the reading tests, the ICC was 0.95 for accentedness and 0.96 for comprehensibility, while for the spontaneous conversation tests, the ICC was 0.91. The final scores for each participant were determined by taking the average of the ratings from both evaluators.

To assess the effectiveness of the ASR technology with peer correction on Chinese EFL learners’ pronunciation, an analysis of covariance (ANCOVA) was performed with pretest scores as the covariate and posttest scores as the dependent variable. The pretest scores were used to adjust for any initial differences in pronunciation ability between the EG and CG, as assessed by the read-aloud task and spontaneous conversation. Before conducting the ANCOVA, normality assumptions were checked for the dependent variable (posttest scores) using Shapiro–Wilk tests, and homogeneity of regression slopes assumptions were checked using Levene’s tests. The assumptions were met for both tests, indicating that the ANCOVA assumptions were satisfied.

Also, the qualitative data collected through the interviews underwent a rigorous and systematic analysis process to ensure objectivity and trustworthiness. Following Grbich's (2012) guidelines, a thematic analysis approach was employed. Initially, the data was carefully reviewed to identify broad categories that emerged from the participants’ responses. These categories were then further analyzed to uncover subthemes that captured more nuanced aspects of the data. This iterative process facilitated a comprehensive exploration of the qualitative data, ensuring accurate identification and interpretation of key themes.

To enhance the reliability of the analysis, two researchers independently conducted the coding process. This approach allowed for cross-validation of the identified themes and minimized potential biases or subjective interpretations. Any discrepancies or differences in coding were thoroughly discussed and resolved through consensus, ensuring consistency and agreement in the interpretation of the data. Furthermore, to enhance the credibility and trustworthiness of the analysis, an independent expert in the field of second language acquisition and qualitative research reviewed and validated the final themes. This external validation served as an additional quality check, confirming the accuracy and relevance of the identified themes (Connelly, 2016).

The themes derived from the data analysis were integrated into the findings and conclusions, providing rich qualitative evidence that supports and complements the quantitative results. This comprehensive analysis of the qualitative data enhances the overall validity and robustness of the study, strengthening the understanding of the impact of ASR technology on L2 pronunciation and speaking skills.

## Results

### Quantitative results

Table 1 presents the descriptive statistics for the variables of the study. The table displays the means and standard deviations for each dependent variable for both the experimental and control groups. The results indicate that both interventions have increased the dependent variables. The experimental group had higher means in all dependent variables compared to the control group, with the largest difference in Global.Speaking2 (experimental group mean = 5.8048, control group mean = 5.3746). Standard deviations were generally small, indicating that the scores were relatively consistent within each group.

**Table 1:** Descriptive statistics for the variables of the study.

	Group	*n*	Mean	Std. Deviation
Accentedness1	Experimental	29	4.1682	0.46202
	Control	32	4.0805	0.45977
Accentedness2	Experimental	29	5.1185	0.89957
	Control	32	4.4760	0.75415
Comprehensibility1	Experimental	29	3.4254	0.47299
	Control	32	3.4938	0.43490
Comprehensibility2	Experimental	29	4.5936	0.76387
	Control	32	4.0593	0.53065
Spon.Speech1	Experimental	29	4.4808	0.59098
	Control	32	4.3807	0.63087
Spon.Speech2	Experimental	29	4.9252	0.55146
	Control	32	4.5769	0.59245
Global.Speaking1	Experimental	29	4.9281	0.49558
	Control	32	4.8252	0.33499
Global.Speaking2	Experimental	29	5.8048	0.58647
	Control	32	5.3746	0.45784

Descriptive statistics indicated that both interventions increased the dependent variables, but to ascertain which group underwent a more substantial increase, ANCOVA was performed. Specifically, ANCOVA was employed to control for pretest scores and to examine the effect of the experimental treatment on the dependent variables while adjusting for initial group differences.

Table 2 presents the results of an ANCOVA conducted to examine the effect of the automatic speech recognition technology intervention on the accentedness of Chinese EFL learners. The ANCOVA was conducted with Accentedness1 (pre-test scores) as the covariate and Group (experimental vs. control) as the independent variable. The dependent variable is Accentedness2 (post-test scores). ANCOVA results indicated a significant effect of the intervention on Accentedness2 after controlling for Accentedness1 (*F* = 8.935, *p* = 0.004, partial eta squared = 0.133). This suggests that the automatic speech recognition technology intervention has a significant effect on improving the accentedness of Chinese EFL learners, after controlling for pre-test scores.

**Table 2:** ANCOVA results for accentedness.

Source	Type III sum of squares	Df	Mean square	*F*	Sig.	Partial eta squared
Accentedness1	8.455	1	8.455	15.404	0.000	0.210
Group	4.904	1	4.904	8.935	0.004	0.133
Error	31.834	58	0.549			

The ANCOVA results (Table 3) indicate that both Comprehensibility1 and Group significantly influenced the participants’ comprehensibility scores. Specifically, Comprehensibility1 accounted for a significant portion of the variation in the scores (Type III SS = 9.155, df = 1, Mean Square = 9.155, *F* = 33.369, *p* < 0.001*, η*^2^ = 0.365), indicating that the participants’ baseline scores significantly affected their comprehension scores after the intervention. Moreover, the Group variable also significantly influenced the scores (Type III SS = 5.332, df = 1, Mean Square = 5.332, *F* = 19.435, *p* < 0.001, *η^2^* = 0.251), indicating that the automatic speech recognition technology intervention had a significant effect on the participants’ comprehensibility more than the control group instruction.

**Table 3:** ANCOVA results for comprehensibility.

Source	Type III sum of squares	Df	Mean square	*F*	Sig.	Partial eta squared
Comprehensibility1	9.155	1	9.155	33.369	0.000	0.365
Group	5.332	1	5.332	19.435	0.000	0.251
Error	15.912	58	0.274			

Table 4 reports the results of an ANCOVA analysis conducted to examine the effectiveness of automatic speech recognition technology on improving spontaneous speech of Chinese EFL learners. The analysis also revealed a significant main effect of the group with a Type III sum of squares of 1.148 (df = 1, 58), a mean square of 1.148, and an *F*-value of 8.599 (*p* = 0.005, partial eta squared = 0.129). This indicates that the group that received the automatic speech recognition technology intervention showed a significantly greater improvement in spontaneous speech than the control group.

**Table 4:** ANCOVA results for spontaneous speech.

Source	Type III sum of squares	Df	Mean square	*F*	Sig.	Partial eta squared
Spon.Speech1	11.650	1	11.650	87.233	0.000	0.601
Group	1.148	1	1.148	8.599	0.005	0.129
Error	7.746	58	0.134			

Table 5 presents the results of the ANCOVA conducted on the global speaking scores of the experimental and control groups. The table shows the sources of variation, including Global.Speaking1 (pretest), Group (experimental vs. control), and Error (within-group variation). The results indicate that there was a significant main effect of group on posttest scores, *F* (1, 58) = 12.401, *p* = 0.001, partial eta squared = 0.176. These results suggest that both interventions were effective in improving the global speaking scores of the Chinese EFL learners, and that the experimental group demonstrated a substantially higher gains than the control group.

**Table 5:** Results for global speaking.

Source	Type III sum of squares	Df	Mean square	*F*	Sig.	Partial eta squared
Global.Speaking1	8.060	1	8.060	57.932	0.000	0.500
Group	1.725	1	1.725	12.401	0.001	0.176
Error	8.069	58	0.139			

#### Qualitative results

The qualitative phase aimed to explore the perceptions and experiences of Chinese EFL learners regarding the use of ASR with peer correction to enhance their L2 pronunciation and speaking performance. The qualitative data obtained from the semi-structured interviews were analyzed thematically, revealing significant themes as summarized below. In this section, we present the major themes along with representative excerpts from participants. Excerpts are labeled with participant identifiers (e.g., P1, P2) for clarity and reference.

##### Theme 1: perceived effectiveness of ASR

Six participants acknowledged the ASR technology as an effective tool for improving their pronunciation. Notably, participants highlighted the accuracy of the ASR technology in detecting errors that they were previously unaware of. For instance, P1 stated, “I found the ASR technology to be really helpful in identifying my mistakes in pronunciation. It was more accurate than relying on just my own ear or my teacher’s feedback.” Additionally, some participants expressed initial skepticism but experienced positive outcomes after using the technology, as observed by P2 who noted, “At first, I was a bit skeptical about using technology to improve my speaking skills, but after using ASR for a few sessions, I could see a noticeable improvement in my pronunciation.”

##### Theme 2: motivation and engagement

Four learners reported that the ASR technology offered a more enjoyable and motivating approach to practicing pronunciation compared to traditional classroom methods. They appreciated the game-like aspect of the technology, as described by P3, who stated, “It made practicing my pronunciation more enjoyable than just doing drills in class.” Furthermore, the ability to track progress served as a source of motivation, as expressed by P4, who mentioned, “It gave me a sense of accomplishment and motivated me to keep practicing.”

##### Theme 3: increased self-awareness

Four participants reported that the use of ASR technology heightened their awareness of their own pronunciation errors, which in turn motivated them to improve. P5 shared, “I never realized how often I mispronounce certain sounds until I started using the ASR software. It’s helped me to be more self-aware and focused on improving my pronunciation.”

##### Theme 4: improved accuracy

Three individuals recognized that the ASR software detected pronunciation errors that were overlooked by human evaluators, resulting in more accurate feedback. P6 expressed surprise, stating, “I was surprised to discover how many errors the ASR software detected that my teacher missed. It aided me in improving my pronunciation in ways I would not have been able to accomplish on my own.”

##### Theme 5: tailored feedback

Four participants appreciated the ASR software for providing personalized feedback on specific pronunciation errors, enabling them to focus on improving those areas. P7 mentioned, “The ASR software was incredibly useful in pinpointing specific sounds that I was having trouble with. It provided me with more customized feedback than my teacher, allowing me to focus on those areas and make greater progress.”

##### Theme 6: increased practice opportunities

The use of ASR technology offered participants more opportunities to practice pronunciation in a low-pressure environment. Three participants reported feeling less nervous compared to being in front of their teacher, as noted by P8, who remarked, “I enjoyed practicing my pronunciation with the ASR software since I felt less nervous than I would in front of my teacher. It gave me more opportunities to practice without feeling self-conscious.”

In sum, the findings from the qualitative phase of this study suggest that the use of ASR technology with peer correction can be an effective tool for enhancing the pronunciation and speaking performance of Chinese EFL learners. Participants perceived the technology as accurate, motivating, and providing tailored feedback that allowed them to focus on improving specific pronunciation errors. The use of ASR technology also increased participants’ self-awareness and provided them with more opportunities to practice their pronunciation in a low-pressure environment.

## Discussion

The current research sought to examine the effect of automatic speech recognition technology on developing the pronunciation and speaking skills of EFL learners. It was concluded that ASR technologies had significant effects on EFL participants’ pronunciation and speaking abilities.

Firstly, it was found that ASR-based instruction enhanced the L2 pronunciation of the EFL participants. This finding is consistent with the findings of other studies that highlight the significant effect of ASR technology on improving pronunciation (Cucchiarini et al., 2009; McCrocklin, 2019a,b; Garcia et al., 2020; Inceoglu et al., 2020, 2023; Yenkimaleki et al., 2021; Cámara-Arenas et al., 2023). Therefore, it can be concluded that is useful for enhancing pronunciation of EFL learners. The use of technology has been found to be effective in improving L2 pronunciation, as it provides learners with instant feedback on their speech production, which allows them to identify and correct errors more efficiently (Foote and McDonough, 2017). ASR allows for a more natural and intuitive interaction between the learner and the technology. Also, it can recognize and analyze speech patterns in real-time, providing immediate feedback to the learner. As a result, learners can focus on producing more accurate and comprehensible speech, which can improve their overall communicative competence. Moreover, the utilization of ASR technology in the context of L2 pronunciation instruction has been found to yield positive effects on learners’ confidence and motivation. By offering learners a sense of control over their learning process, ASR technology empowers them to actively engage in their pronunciation development (Liakin et al., 2015; O’Brien et al., 2018; Tseng et al., 2022). The enhanced confidence and motivation experienced by learners when utilizing ASR technology can lead to increased engagement and persistence in their L2 learning endeavors (Golonka et al., 2014). This increased engagement and persistence are crucial factors contributing to improved language proficiency outcomes (Jayalath and Esichaikul, 2022). Learners who feel empowered and in control of their pronunciation practice are more likely to invest time and effort into refining their speaking skills (Rahimi and Fathi, 2022).

Having provided learners with instant feedback and targeted error detection, ASR technology enables learners to identify and address their pronunciation errors more effectively (McCrocklin, 2016). This personalized feedback not only enhances learners’ self-awareness but also facilitates a deeper understanding of the target language phonetic system. Such insights contribute to the development of more accurate pronunciation skills, thereby fostering overall speaking proficiency (McCrocklin, 2019b). ASR technology adapts to individual learners’ needs, offering personalized feedback and targeted interventions tailored to their specific pronunciation challenges. This individualized approach allows learners to focus on their areas of weakness and allocate their efforts efficiently (Jiang et al., 2023).

Moreover, Liakin et al. (2015) tested ASR, teacher-driven pronunciation feedback, and no feedback strategies on learners’ pronunciation and discovered that only the first strategy enhanced their pronunciation. In harmony with this finding, McCrocklin (2016) concluded that even short-term ASR education can result in significant gains in pupils’ pronouncing skills. In the same vein, Mroz (2018) found that using ASR to practice pronunciation positively helps pupils. Besides, Liu et al. (2019) asserted that the ASR dictation technique was useful, with an improvement in the English pronunciation of Chinese-speaking pupils, which supports the finding of the present study.

The second finding of the present study was that ASR-based instruction improved the speaking skills of the EFL participants. This finding is in line with previous studies that emphasized the critical role of ASR technologies in improving L2 speaking skills (Chen, 2017; Evers and Chen, 2021; Bashori et al., 2022; Lai and Chen, 2022; Jiang et al., 2023). These research studies have demonstrated that ASR technology may be a valuable tool for boosting the speaking skills of FL learners. According to the Interactionist Hypothesis (Long, 1996), language learning occurs through interaction, which involves learners receiving feedback on their linguistic output. ASR technology can provide immediate and accurate feedback on the pronunciation, intonation, stress, and rhythm of learners’ speech, which is crucial for improving their speaking performance. Furthermore, the use of automatic speech recognition technology in language learning is supported by the Cognitive Load Theory (Sweller, 1988), which suggests that learners have limited cognitive resources and that extraneous cognitive load should be minimized to facilitate learning. By providing automated feedback on learners’ speech, ASR technology reduces the cognitive load associated with receiving feedback from teachers, allowing learners to focus on their linguistic production. The finding also aligns with the research on the effectiveness of CALL in promoting L2 speaking skills (Blake, 2017; Cardoso, 2022). ASR technology provides a more accurate and objective assessment of learners’ speaking performance than traditional methods of assessment, such as teacher feedback or self-evaluation (Kim, 2006).

One possible explanation for this assertion is that engaging in speech activities through ASR can enhance a person’s motivation to participate in speaking activities in a second or foreign language (Inceoglu et al., 2020). Additionally, it has been found that learners value such systems, and the feedback provided can be valuable in improving the pronunciation of challenging speech sounds. As mentioned earlier, the use of ASR technology leads to significant improvements in learners’ pronunciation (Inceoglu et al., 2023). As a result of improved pronunciation, individuals may experience increased motivation and enthusiasm to engage in speech activities, leading to an overall enhancement in their communication competence. This finding is consistent with the assertion by Brinton et al. (2010) that pronunciation plays a crucial role in foreign language communication as it ensures speech comprehensibility to others.

Concerning the second research question, the qualitative findings revealed participants’ perceptions of increased motivation and engagement when utilizing ASR technology as an intervention for improving their pronunciation and speaking skills. Participants reported that the accuracy of the ASR technology in detecting errors, which they were previously unaware of, contributed to heightened self-awareness and a motivation to improve their pronunciation. This finding suggests that the technology served as a tool for enhancing learners’ metalinguistic awareness and fostering a sense of personal responsibility for their language development. Moreover, participants expressed that the game-like features of the technology and the ability to track their progress offered a more enjoyable and motivating alternative to traditional classroom methods. This observation aligns with the existing literature on gamification, which has demonstrated its potential to increase motivation and engagement in learning contexts (Jayalath and Esichaikul, 2022). It is worth noting that the benefits of ASR technology in providing more accurate and objective feedback compared to human evaluators were also highlighted by participants. The technology’s ability to detect pronunciation errors that may have been missed by human evaluators suggests its potential to offer learners more comprehensive and targeted feedback. This finding resonates with prior research demonstrating the advantages of ASR technology in providing feedback within language learning contexts (Ling and Chen, 2023). Furthermore, participants emphasized the personalized feedback provided by the ASR software, enabling them to concentrate on improving specific areas of their pronunciation. This personalized approach aligns with research indicating that tailored feedback enhances the effectiveness of L2 learning (Fathi and Rahimi, 2022; Pérez-Segura et al., 2022). Finally, participants noted that the use of ASR technology created a low-pressure environment that afforded them more opportunities to practice their pronunciation. This finding corresponds with previous research, which has demonstrated the benefits of utilizing technology to provide learners with additional practice opportunities (Peng et al., 2021).

Overall, the findings can be interpreted in light of Vygotsky’s (1978) SCT framework. From a socio-cultural theory perspective, learning is seen as a social and collaborative process that takes place through meaningful interactions with others in the context of shared activities and goals (Vygotsky, 1986). The role of technology in mediating these interactions and creating opportunities for learning is justified based on SCT (Ma, 2017). In light of these ideas, the finding that automatic speech recognition technology enhanced L2 pronunciation and speaking performance of EFL students can be explained in several ways. First, technology provides a novel and engaging way for learners to interact with language and receive feedback on their performance (Levis, 2007). Having used ASR technology, learners receive instant feedback on their pronunciation, allowing them to correct errors and improve their accuracy (Evers and Chen, 2022). This process can increase learner motivation and engagement with the learning task, leading to more effective learning outcomes. Second, technology-mediated learning can create opportunities for learners to interact with authentic language input and engage in real-world communicative tasks (Ziegler, 2016). ASR technology can provide learners with access to authentic speech samples and real-world scenarios in which they must use their language skills to communicate effectively. This exposure to authentic language input can improve learners’ comprehension and production skills by providing them with a rich and varied source of input and feedback. Third, technology can facilitate collaborative and social learning by connecting learners with peers and teachers in virtual learning environments (Kukulska-Hulme and Viberg, 2018). By using online platforms and tools, learners can communicate with one another, share resources, and receive feedback from teachers and peers in real-time (Lenkaitis, 2020). This collaborative learning process can enhance learners’ communicative competence by providing opportunities for meaningful interaction and negotiation of meaning (Levy, 2009).

## Conclusion

The present study aimed at investigating the effect of automatic speech recognition technology in improving the pronunciation and speaking skills of EFL learners. This study indicated that the use of automatic speech recognition technology with peer correction is an effective tool for improving L2 pronunciation and speaking skills of Chinese EFL learners. The results of the quantitative analysis show that ASR helped EFL participants to enhance their L2 pronunciation and demonstrated significant greater improvements in global speaking skill. The qualitative analysis of student feedback also revealed that the majority of participants found the ASR technology helpful in improving their L2 pronunciation and speaking skills.

According to the study’s results, it is advised that ASR be included in English language curriculum programs in schools. ASR technology with peer correction can be used as a supplementary tool in language classrooms to enhance L2 pronunciation and speaking skills. It can also be integrated into language learning software to provide learners with additional practice opportunities outside the classroom. The voice recognition approach may be used in other English language classrooms at various academic levels. Additionally, English language instructors may be educated to utilize ASR to reinforce pronunciation. The incorporation of ASR into educational and instructional settings should be prioritized. Speech recognition systems need to be set up and utilized as crucial instruments in the learning process when utilizing a computer and the Internet. More study is required in the domain of ASR-based pronunciation instruction. Finally, investigators may undertake comparable studies with different classifications, larger samples, and various procedures and techniques.

It must highlight that when ASR technology is deployed in a classroom, specific obstacles arise; that was likewise obvious in the current study. ASR dictation tools have significant limitations when it comes to pronunciation training. Such ASR technologies can provide a lot of practice and rapid feedback, but they do not have any capabilities linked to phonetic representations. They lack capabilities that describe how to employ vocal apparatus for specific sounds, or how the intended sounds vary from the individuals’ native language. Students require greater assistance with their pronunciation and understanding. Despite major advancements in ASR platforms, they still have inferior identification accuracy when contrasted with a human assessment approach, particularly in a noisy setting (Loukina et al., 2017). As a result, several students have expressed moderate dissatisfaction and displeasure with the software’s recognition skills. Further study might look into how individuals and teams can work together to solve this challenge. For instance, students’ annoyance may be decreased if they can exchange comments on each other’s pronunciation, which is more realistic than digital feedback. Because these findings might be attributed to a variety of causes, additional research into accentedness is required. Investigators, for instance, may integrate ASR technologies with native speaker teaching or employ various forms of ASR technology. According to findings, it is critical for further research to use ASR-based web experiments to learn which elements are useful and how much time pupils need to commit. It also requires longer-term trials with a diverse range of participants.

In summary, the findings of this study provide evidence supporting the value of integrating ASR technology with peer correction as a means to facilitate the development of L2 learners’ pronunciation skills and enhance their overall speaking proficiency. However, it is crucial to acknowledge the limitations inherent in this investigation. First and foremost, it is important to recognize that the research was conducted exclusively with a specific group of intermediate-level Chinese EFL learners. As a result, caution must be exercised when generalizing the findings to other populations or proficiency levels. Future studies should endeavor to incorporate a more diverse range of participants, thus augmenting the external validity of the research outcomes. Secondly, the study was constrained by a relatively brief intervention period, which may have influenced the extent of the observed improvements. To gain a more comprehensive understanding of the sustainability and long-term effects of incorporating ASR technology with peer correction, longitudinal investigations should be pursued. Such studies would provide valuable insights into the enduring impact of this intervention on L2 pronunciation and speaking skills. In addition, it is important to acknowledge that the qualitative analysis of participant perceptions was based on self-reported data, introducing potential biases and subjectivity. To bolster the robustness of the findings and mitigate reliance on self-reported information, the inclusion of additional objective measures, such as independent assessments conducted by experts or objective evaluations of pronunciation, would be advantageous. Finally, although efforts were made to account for learners’ background knowledge, it is essential to acknowledge that the influence of participants’ prior language exposure, educational backgrounds, and other individual factors on their speaking proficiency might still exist, which is a common consideration in studies examining L2 speaking skills. As such, future studies could consider incorporating measures to assess participants’ background knowledge. This could be achieved through pre-assessment surveys or interviews that gather information about their educational background, language learning experiences, exposure to the target language, and familiarity with the assessment topics.

## Data availability statement

The data of this article will be made available by the authors upon request. Requests to access these datasets should be directed to WS, sunwn402@nenu.edu.cn.

## Ethics statement

The studies involving human participants were reviewed and approved by School of Foreign Languages, Changchun Institute of Technology, Changchun. The patients/participants provided their written informed consent to participate in this study.

## Author contributions

The author confirms being the sole contributor of this work and has approved it for publication.

## Funding

The research is supported by 2022 Jilin Provincial Research Projects in higher education: Exploration and Practice of Core Literacy in College English Flipped Class [Project No: JGJX2022C90].

## Conflict of interest

The author declares that the research was conducted in the absence of any commercial or financial relationships that could be construed as a potential conflict of interest.

## Publisher’s note

All claims expressed in this article are solely those of the authors and do not necessarily represent those of their affiliated organizations, or those of the publisher, the editors and the reviewers. Any product that may be evaluated in this article, or claim that may be made by its manufacturer, is not guaranteed or endorsed by the publisher.

## Appendix

### Interview questions

1. Can you describe your experience with the ASR technology and peer correction? What did you find most helpful, and what were some challenges you faced?
2. How d o you feel the ASR technology and peer correction influenced your L2 pronunciation and speaking skills? Can you provide specific examples?
3. In what ways did the ASR technology and peer correction differ from traditional teacher-led feedback and instruction? Which method did you find more effective, and why?
4. How did the ASR technology and peer correction impact your overall confidence in speaking English? Did you feel more comfortable speaking after using this technology?
5. Can you describe any changes or improvements you noticed in your L2 pronunciation and speaking skills after using the ASR technology with peer correction? Did you feel more confident or accurate in your pronunciation and speaking abilities?
